# Current Status of Fasciolosis of Goat in Sylhet, Bangladesh: An Integrated Morphomolecular Study

**DOI:** 10.1155/2022/6159388

**Published:** 2022-09-09

**Authors:** Chamali Akter Shykat, Saiful Islam, Foyez Ahmed, Kazi Mehetazul Islam, Jamal Uddin Bhuiyan, Tilak Chandra Nath

**Affiliations:** ^1^Department of Parasitology, Sylhet Agricultural University, Sylhet, 3100, Bangladesh; ^2^Parasite Resource Bank, Bangladesh

## Abstract

Epidemiological information and proper identification of *Fasciola* species present in Bangladesh are important for control. This study was aimed at determining the prevalence of liver fluke infection of goats in Sylhet, Bangladesh, and identifying those using integrated morphometric and molecular techniques. A total of 260 slaughtered goats (*Capra hircus*) were examined, and flukes were collected from infected liver using sterilized forceps. Fasciolosis prevalence in goats was 35.38% (92/260) across all age and sex categories. Female goats were found more infected (37.14%, 65/175) than male goats (31.76%, 27/85), while infection rate was found higher in young animals (37.91%, 69/182) compared to adults (29.48% 23/78). Infection rate was observed higher in rainy season (52.96%, 45/85), followed by winter (27.38%, 26/95) and summer (26.25%, 21/80). Collected flukes were examined by light microscopy after being stained with Semichon's acetocarmine, and sequences of mtDNA *Cox*1 genes were obtained. Ten adult flukes were measured, 38.72 ± 3.37 mm in length and 11.8 ± 1.9 mm in width. Based on morphometric features especially branching of the testis and body length/body width ratios (3.28 ± 0.42), the flukes were primarily identified as *Fasciola gigantica*. Amplicon sequences were compared by BLAST and the *cox*1 sequences showed 97.1-99.3% similarity with the reference sequences (*F. gigantica* and *Fasciola* sp.) from GenBank. In this study, we found a considerable prevalence of fascioliasis in goats, and *F. gigantica* was solely identified with variation. To control these parasites and prevent potential public health risks, appropriate control techniques must be developed.

## 1. Introduction

Goat is a major source of nutrition, contributing halfway to consumable meat production in Bangladesh. Rearing goats are an integral part of the rural economy and play an important role in the livestock industry. They are highly profitable because of their short generation interval and their products (milk, meat, and skin) are easily marketable [1, 2]. Goats are often farmed by poor farmers and disadvantaged women in Bangladesh with very little financial input and are well recognized as a strategy for poverty alleviation [3]. Despite the vast population of goats in Bangladesh, productivity is low owing to management issues and the presence of animal diseases. Asian Development Bank estimated the loss of generation and reproductive rate of animal parasites to the extent of 50% in Bangladesh [4]. Due to the lack of proper veterinary care, conventional husbandry practices, and development of anthelmintic resistance, parasitism is still considered one of the significant problems causing considerable losses in goat production [3].

Fascioliasis or liver fluke infections in food animals bear considerable economic and public health importance. The disease is most widely disseminated in tropical and subtropical countries, with cases documented in more than 50 countries, especially in Asia, Africa, and America [5]. High prevalence of fascioliasis is usually observed in low-income communities due to the intimate relationships between livestock and human. Bangladesh is endemic to caprine fascioliasis; the prevalence of fascioliasis in live animals has been reported to vary from 10 to 32% in live goats and 3.8–22% in slaughtered goats. In neighboring countries, caprine fascioliasis was reported 2.35–15% in India and 4.08–28.75% in Pakistan [6]. *Fasciola hepatica* (mostly found in temperate zones) and *Fasciola gigantica* (found in tropical zones) are two well-known *Fasciola* species that cause fasciolosis. *Fasciola* infects the livers and bile ducts of ruminants and other mammals, and Lymnaeidae snails serve as intermediate hosts. Over 180 million humans are estimated at risk of zoonotic infection; they make the disease a great public health concern [7]. The cost of animal production losses due to fasciolosis is estimated to be $200 million per year worldwide [8]. Even when animals appear to be healthy, livestock suffering from subclinical fasciolosis can have significant production losses [9]. Fascioliasis is a global problem, however more prevalent in Bangladesh due to the presence of a diverse variety of agroecological factors that are preferable for intermediate host and parasite species. Despite the adverse effects on the livestock sector and public health safety, little is known about the dynamics of *Fasciola* species in Sylhet, Bangladesh. Hence, the present work is aimed at determining the prevalence of liver flukes in slaughtered goats in Sylhet, Bangladesh, identifying those using combined morphomolecular method, and correlating the results with the presence of liver parasites regarding age, sex, and season.

## 2. Materials and Methods

### 2.1. Sample Collection and Morphological Analysis

Sample collection was carried out in and around Sylhet City Corporation of Bangladesh during 2018 and 2019. Flukes were collected from the liver of slaughtered goats (*Capra hircus*) and preserved separately in 70% ethanol and 10% formalin for molecular and morphological observations. Sylhet region is located in the northeastern part of Bangladesh, geographically located between 24.8917°N and 91.8833°E. The tropical and monsoon climates of this region facilitate the growth of parasites. Morphological examinations were conducted at the Department of Parasitology, Sylhet Agricultural University, Bangladesh, and molecular analysis was done at the Department of Parasitology, Chungbuk National University, South Korea. Parasite specimens were deposited in the Parasite Resource Bank, Bangladesh (PRB_SR_000005-14) for future use and will be available with releasable requests to the corresponding author. Formalin-fixed worms were processed for morphological examination. Flattened specimens were stained with Semichon's acetocarmine. Before mounting, a graded ethanol series (70, 80, 90, 95, 100, and 100%), 50% xylene and 50% absolute ethanol, and 100% xylene were used for dehydration and clarifying. The specimens were examined using a light microscope (Olympus BX-53, Japan), and all organs were measured using an ocular micrometer.

### 2.2. PCR Amplification and Sequence Analysis

DNA extraction was done from ethanol-preserved samples following a previous protocol [4, 10]. Three adult flukes collected from separate host animals were washed three times in PBS before DNA extraction. Genomic DNA from the posterior part of each fluke, excluding the uteri and ootype, was extracted by using an available commercial kit (DNeasy Blood and Tissue Kit, Qiagen, Hilden, Germany; Cat Nos. 69504 and 69506). Except for the elution step, where distilled water was used instead of elution buffer and was repeated twice, the remaining DNA extraction was performed according to the manufacturer's protocol. The concentration and purity of DNA were measured (NanoDrop Spectrophotometer, Thermo Fisher Scientific Solutions Co., Ltd., Korea) and stored at –20°C until required for polymerase chain reaction (PCR). A region within mitochondrial cytochrome oxidase *c* subunit I (*cox*1) was amplified and sequenced by cycle sequencing. PCR was performed with previously described primers HCO2198 (5′-ggtcaacaaatcataaagatattgg-3′) and HCO2198 (5′-taaacttcagggtgaccaaaaaatca-3′) . PCR amplification was carried out in a final reaction mixture containing 25 *μ*L of PCR mix including 1 *μ*L of each primer (10 pmol), 1 *μ*L of each sample DNA, 6 *μ*L of 5X PCR Master Mix (ELPIS biotech, South Korea), and 16 *μ*L of nuclease-free distilled water. A negative control (distilled water) was applied in each run. PCR amplification was preceded by a 5 min initial denaturation step at 95°C, followed by 35 cycles of 1 min each at 94°C for denaturation, 48°C for annealing, and 72°C for extension. These cycles were followed by a 5 min final extension step at 72°C. The PCR products were run on a 1.5% agarose gel and visualized using a UV transilluminator. The amplified PCR products were then purified using DokDo-Prep PCR Purification Kit (ELPIS biotech, South Korea). All obtained sequences were aligned with Clustal W and Bioedit software version 7.1. Analysis of sequencing data was carried out using the National Center for Biotechnology Information BLAST program and database (http://www.ncbi.nlm.nih.gov/) and compared with the sequences present in GenBank database. Multiple sequence alignments were performed with Geneious software version 9 (Biomatters, New Zealand). The multiple alignments were performed with the program Muscle [11] implemented in MEGA7 software [12].

### 2.3. Statistical Analysis

Statistical analysis was carried out using the statistical software SPSS (version 15.2) and Microsoft Excel 2010. Prevalence was determined as the number of individual animals infected with flukes per total number of animals screened. Association of fasciolosis with age, sexes, and season was determined using chi-square test (*χ*^2^), and values of *p* ≤ 0.05 were considered as significant.

## 3. Results and Discussion

The livers of 260 slaughtered goats were investigated, and 92 (35.38%) were confirmed to be infected with the liver fluke (Table 1). Infection was more common in younger goats than in the older. Females had a greater prevalence (37.14%) than males (31.76%). The seasonal prevalence of *Fasciola* infection in goats was detected in the rainy (52.96%), winter (27.38%), and summer (26.25%) seasons in the current study (Table 1).

**Figure 1:**
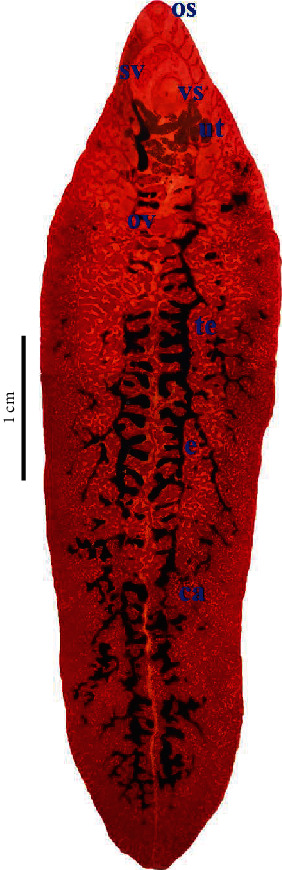
Adult *Fasciola gigantica* (Semichon's acetocarmine stain); oral sucker (os), seminal vesicle (sv), ventral sucker (vs), uterus (ut), ovary (ov), testis (te), and caecum (ca).

In epidemiological investigations of parasite zoonoses, detailed knowledge on the morphological and molecular characteristics of parasite species is crucial. Adult worm specimens were precisely identified in this study using morphological characteristics and morphometrical analyses. The worms were described using whole-mounted specimens of adult worms (Figure 1).  Table 2 shows the morphometric measures (mean ± standard deviation) of the flukes studied. Shoulder was less prominent. Sperm was seen in the seminal vesicle. The morphological observation and morphometric features of the present specimens were consistent with the description of *Fasciola gigantica* documented previously [5, 13]. According to their morphometric traits, *Fasciola* species have typically been divided in the literature. *F. gigantica* was distinguished from *F. hepatica* by its long and slender body, while *F. hepatica* was typically shorter and had wide shoulders [5]. Our research revealed that an L/W value of >3.00 is necessary to distinguish between the two species. However, morphometric features of the fluke body sections can differ according to the host species, the severity of the infection, and crowding effects like host reactivity and age resistance. Thus, morphological approaches for differentiating *Fasciola* species, particularly *F. gigantica* and *F. hepatica*, can be unreliable [13].

**Figure 2:**
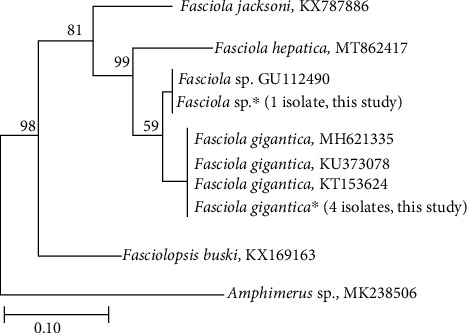
Phylogenetic tree of *Fasciola* spp. inferred from *cox1* gene sequences reconstructed using the maximum likelihood (ML) method. Bootstrap values are shown on the nodes. The scale bar represents 0.10% divergence.

Molecular studies provide insight into the biology and phylogenetic relationship between different parasite species. We used the mitochondrial cox1 (mtDNA cox1) gene to determine the current species accurately. It is difficult to distinguish fluke species with similar morphological structures or minor morphological variations. A combined method, including both morphological and DNA-based molecular techniques, should provide a more reliable means of identification. Representative specimens were taken and exposed to molecular assessment to confirm and analyze the genetic variation of helminth species. In our study, the *cox*1 sequence identities of *Fasciola* species ranged from 99.4% to 99.6%, when compared with reference sequences from GenBank database using BLAST search (http://www.ncbi.nlm.nih.gov/BLAST/). The PCR with the HCO2198 and HCO2198 primers generated approximately 548 bp of the product. After trimming and sequence alignment, the isolates from this investigation are clustered with *F. gigantica* (MH621335, KU373078, KT153624, and KT153624) and clearly distinct from *F. hepatica* (MT862417), according to a phylogenic tree created using the maximum likelihood (ML) method. Interestingly, one specimen was clustered with *Fasciola* sp. GU112490 (Figure 2). High haplotype diversity was observed from the *F. gigantica* population in Bangladesh by Mohanta et al. [4], and they suggested differentiating aspermic *Fasciola* sp. from other *Fasciola* species. Therefore, to fully comprehend the evolutionary history of this species, additional genetic research on *Fasciola* flukes from other hosts and utilizing various markers is required.

The current study observed an increased prevalence of fascioliasis in goats than Tasawar et al. [14], Hossain et al. [15], and Akhtar et al. [16], where it reported 28.8%, 20.7%, and 12.5%, respectively. This increased prevalence of liver flukes and their potential of transmission may be related to the livestock management system, low-laying grassland, and the usage of contaminated water resources. Our results were consistent with those of Isah [17], who found that 35.0% of goats had fascioliasis by examination of bile samples from livers. Higher infection (42.4%) was also recorded in West Gojjam, Amhara region, by Bogale et al. [18]. Environmental and climatic parameters (temperature, rainfall, and humidity), management methods, absence of deworming, low laying, grassland or overgrazing, housing system, sample size, presence of intermediate hosts, sex, age, breed, season, and owner illiteracy all play a vital role in parasite transmission in animals. The worm's population is also influenced by the availability, abundance, and spread of their intermediate host snails. Fascioliasis is more common in young animals (37.8%) than in adults (29.5%). The natural discharge of adult worms from the intestine with enhanced resistance and immunity may account for the drop in the percentage rate as animals get older [19]. In addition, the higher prevalence in young animals might be attributed to a lack of immunity, which plays a big role in parasite proliferation and growth.

In this study, it was found that female goats were highly at risk of *F. gigantica* infection in comparison with the male. Hassan et al. [20] also observed the higher prevalence in females compared to male goats. It might be because females' immunity to diseases is lowered by stress factors like such as calving and lactation. During the study, samples were collected in different months and were divided according to season, viz., rainy (July to October), winter (November to February), and summer (March-June). The current study showed that the highest infection was observed in the rainy season while light infection in the summer season at the study area. The seasonal prevalence of fasciolosis in the liver of slaughtered goats was significantly higher in rainy followed by winter and summer seasons, and these results are in agreement with Hossain et al. [15], Haridwal et al. [21], and Yemisrach and Mekonnen [22]. This might be due to the climatic conditions (rainfall, temperature, and humidity) and the availability of an intermediate hosts (snails) during this season. During the rainy season, some nematodes developed their exogenous stages as well as increased their larval population. There are a few limitations to our study. First, the sample size of this study was small to find some relationship or differences in statistical power. Second, we did not collect feces samples, making it impossible to describe egg characteristics. Therefore, further epidemiological studies with a hierarchical sample approach are required.

## 4. Conclusion

In this study, a substantial prevalence of fascioliasis was found in slaughtered goats in Sylhet, Bangladesh. Collected flukes were identified as *F. gigantica*-based morphological features, however variation was observed in mtDNA *cox*1 gene analysis. Further studies are required to clarify this issue. Fasciolosis in goats mostly occurred in the rainy season, and younger and female goats were also found more susceptible. To mitigate the possible economic loss, preventive techniques such as the strategic use of anthelmintics, lowering of intermediate hosts, and appropriate farming operations may be advised. The results of this study will serve as a supportive data for further molecular profiling and comparison with other fluke species from different regions of Bangladesh. The study might be helpful to the policymaker to take effective preventive and control measures against this fluke.

## Figures and Tables

**Table 1:** Overall prevalence and association of fasciolosis between age, sex, and season.

Variables		No. of examined	No. of positive	Prevalence (%)	*p* value
Overall prevalence		260	92	35.38	—
Age	Young (<1.5 years)	182	69	37.91	0.3621
	Adult (≥1.5 years)	78	23	29.48	
Sex	Male	85	27	31.76	0.5539
	Female	175	65	37.14	
Season	Rainy	85	45	52.96	0.3708
	Winter	95	26	27.38	
	Summer	80	21	26.25	

**Table 2:** Morphometric measures (mean ± SD) of liver flukes collected from slaughtered goats (*n* = 10).

Host	Body size (mm)^∗^						
	BL	BW	DBS	LCC	WCC	DBS	BL/BW
Goat	38.72 ± 3.37	11.8 ± 1.9	1.62 ± 0.19	2.35 ± 0.42	2.74 ± 0.63	1.78 ± 0.21	2.56 ± 0.42

^∗^BL: body length; BW: body width; BL/BW: ratio of body length to body width; DBS: distance between suckers; LCC: length of cervical cone; WCC: width of cervical cone; DBVE: distance between oral and ventral suckers; BL/BW: Ratio between body length and body width.

## Data Availability

The samples used for the current study will be available from the corresponding author and Parasite Resource Bank, Bangladesh, on reasonable request.

## Abbreviations

PCR: Polymerase chain reaction
mtDNA: Mitochondrial deoxyribonucleic acid
*Cox*1: Cytochrome *c* oxidase I
BP: Base pair
BLAST: Basic local alignment search tool
WHO: World Health Organization.
